# Sex and menstrual cycle influence human spatial navigation strategies and performance

**DOI:** 10.1038/s41598-023-41153-x

**Published:** 2023-09-11

**Authors:** Alana Brown, Ford Burles, Giuseppe Iaria, Gillian Einstein, Morris Moscovitch

**Affiliations:** 1 Psychology, University of Toronto, 100 St. George Street, Toronto, ON M5S 3G3 Canada; 2 Department of Psychology, Hotchkiss Brain Institute, and Alberta Children’s Hospital Research Institute, University of Calgary, Calgary, AB T2N 1N4 Canada; 3 Rotman Research Institute, Baycrest Academy of Research and Education, Baycrest Health Sciences, Toronto, ON M6A 2E1 Canada; 4 Linköping University, 581 83 Linköping, Sweden

**Keywords:** Psychology, Human behaviour

## Abstract

Which facets of human spatial navigation do sex and menstrual cycle influence? To answer this question, a cross-sectional online study of reproductive age women and men was conducted in which participants were asked to demonstrate and self-report their spatial navigation skills and strategies. Participants self-reported their sex and current menstrual phase [early follicular (EF), late follicular/periovulatory (PO), and mid/late luteal (ML)], and completed a series of questionnaires and tasks measuring self-reported navigation strategy use, topographical memory, cognitive map formation, face recognition, and path integration. We found that sex influenced self-reported use of cognitive map- and scene-based strategies, face recognition, and path integration. Menstrual phase moderated the influence of sex: compared to men, women had better face recognition and worse path integration, *but only during the PO phase*; PO women were also better at path integration in the presence of a landmark compared to EF + ML women and men. These findings provide evidence that human spatial navigation varies with the menstrual cycle and suggest that sensitivity of the entorhinal cortex and longitudinal axis of the hippocampus to differential hormonal effects may account for this variation.

## Introduction

Compared to women, men show performance advantages in some spatial navigation tasks^1^. However, these sex differences may depend on hormone levels (i.e., 17β-estradiol (E2) and progesterone) varying across the menstrual cycle. Despite evidence of neural changes with the menstrual cycle^2^, the functional effects of such changes on navigation have rarely been assessed across multiple cognitive domains and task types, with most research focusing on only one dimension. Thus, the current study asks whether sex differences in various facets of medial temporal lobe-dependent navigation depend on menstrual phase.

Numerous cognitive processes and neural networks underlie spatial navigation; assessing different cognitive domains allows for a more complete understanding of these processes. Successful navigation depends, in part, on cognitive maps, namely, mental representations of the environment, including landmarks and their relative locations^3–5^. Three types of knowledge are needed to form/use cognitive maps: (1) Landmark knowledge—memory for objects/features in the environment, (2) Route knowledge—memory for directional sequences, such as “right, left, then straight”, and (3) Survey knowledge—memory for metric information about the location and estimated distances between landmarks, independent of one’s position^6,7^. Survey knowledge particularly helps build a cognitive map of the environment^8^. These types of knowledge can be organized either in self-centered (egocentric) or viewpoint-invariant (allocentric) reference frames^9^. Also critical for navigation is path integration, involving the continual idiothetic integration (relating to use of internal rather than external cues) of location information to estimate current position with respect to a starting location^10^.

A crucial brain region implicated in supporting cognitive map formation is the hippocampus^11–15^. The cognitive map-based navigation strategy that survey knowledge subserves is particularly related to increased posterior, relative to anterior, hippocampus volume, suggesting a potential trade‐off between contributions of anterior and posterior hippocampal segments^16^. Brain areas underlying path integration include the hippocampus^11,17,18^ and entorhinal cortex^19,20^, with potentially more dependence on the entorhinal cortex than the hippocampus^21^. Thus, survey knowledge may be posterior hippocampus-dependent^22^, while path integration may be entorhinal cortex-dependent^21^.

**Figure 1:**
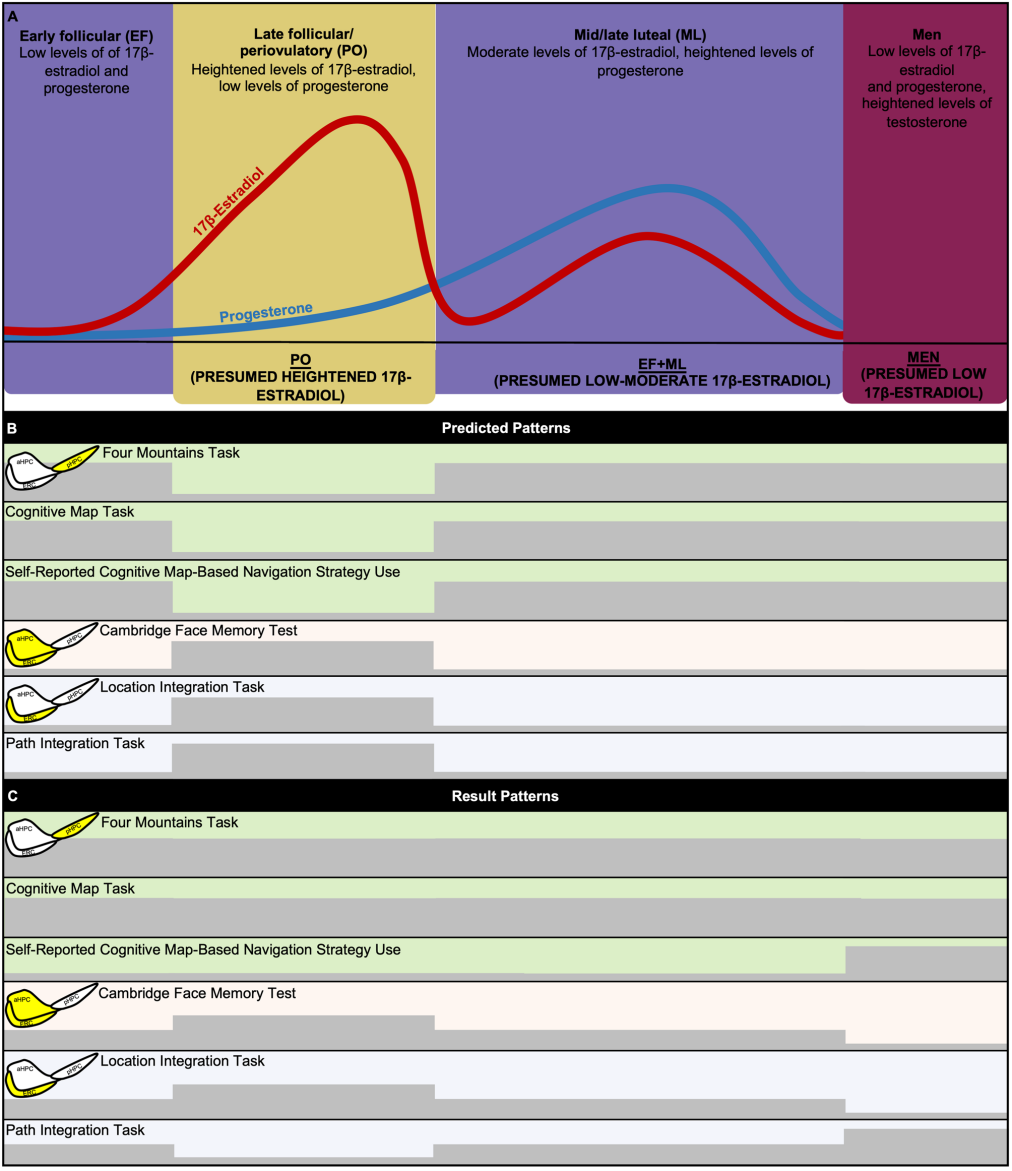
Canonical depiction of menstrual phase and group comparison predictions and results. (**A**) Graphic depicting canonical example of 17β-estradiol and progesterone level fluctuations throughout the menstrual phase and the grouping approximations used for this study; (**B**) Depiction of canonical predicted comparative patterns of task performance and self-reported spatial navigation strategy use for the menstrual phase groups and men; (**C**) Depiction of canonical comparative patterns of results for task performance and self-reported spatial navigation strategy use for the menstrual phase groups and men. Yellow highlight = medial temporal lobe region that may be particularly relevant for the measure. *aHPC* anterior hippocampus, *pHPC* posterior hippocampus, *ERC* entorhinal cortex.

Structure and function of the hippocampus and entorhinal cortex fluctuate with E2 and progesterone levels across the menstrual and estrous cycles in humans and rodents, respectively. Across the human menstrual cycle, both hormones are low during the early follicular (EF) phase, E2 rises and peaks in the late follicular/periovulatory (PO) phase to drop again and rise to moderate levels during the mid-late luteal (ML) phase—when progesterone is at its peak (Fig. 1A)^23^. Throughout the menstrual cycle, E2 levels have different effects on the hippocampus and entorhinal cortex; in PO, anterior hippocampus volume increases^24^ and E2 is positively associated with bilateral hippocampal connectivity^25^. Across the five-day rodent estrous cycle, circulating E2 is positively related to dendritic spine density of hippocampus cornu ammonis 1 subfield pyramidal cells^26^. E2 may also affect entorhinal cortex structure and function. For example, ovariectomy in rodents leads to disruptions in cholinergic function of the entorhinal cortex^27^. The behavioral effects of such neural changes on navigation have been rarely studied.

There is evidence for sex differences in hippocampus-dependent navigation performance. Men more frequently report use of navigation strategies dependent on survey knowledge and allocentric perspective-taking^28–33^. Women more frequently report use of egocentric route-based strategies^29,32–35^. Men perform best when keeping track of unseen navigational starting points underlying path integration^31^ and following instructions using cardinal directions and metric distances^36^, while women perform best when navigation instructions use landmark-based strategies^36^. Men are also significantly faster than women at reaching a target in a virtual maze^30^, make fewer moves navigating a wire-frame virtual environment^37^, take more shortcuts^34^, and form/use survey knowledge more successfully to create accurate cognitive maps^38,39^. In terms of spatial navigation with parietal cortex-dependent landmark and route knowledge, fewer sex differences are noted^29,36,40^. Thus, when salient landmark information is available during spatial navigation, women perform as well as men^41^. For entorhinal cortex-dependent path integration, there is little agreement regarding sex differences, with some studies reporting no differences^42,43^, and others reporting an advantage for men^39,44^. Broadly, most studies have disregarded how the menstrual cycle may account for variability in noted sex differences.

Given the rapid timeline for steroid hormone-associated medial temporal lobe structural and functional changes, the menstrual cycle provides a unique opportunity to study how these potential alterations relate to navigation performance variability^45^. However, while the current study discusses how behavioural sex and menstrual phase differences relate to medial temporal lobe structure and function, neuroimaging was not used to measure the brain and we do not suggest that brain differences are the *sole cause* of behavioral changes^46^. Instead, these putative brain-behaviour relationships are used to derive predictions about changes in navigation behaviour across the menstrual cycle. Navigation has rarely been studied with respect to menstrual cycling and certainly not with multiple tasks tapping into different spatial functions; therefore, the current study fills an important research gap. While it is commonly reported that men have a visuospatial advantage in mental rotation^47^, it has been shown to be contingent on menstrual phase, with women in the EF phase performing similarly to men, and women in the PO phase performing worse than men^48^. In terms of spatial navigation, during the ML phase, women focus more on landmark information and navigate with greater ease from an egocentric perspective compared to women in the follicular phase^49,50^. Collectively, these studies suggest that hippocampus-dependent spatial abilities may vary with the menstrual cycle.

The hippocampus has different cortical connectivity and functionality along its longitudinal axis^51^, with spatial navigation being more dependent on the posterior hippocampus, and face recognition being more dependent on the anterior hippocampus and entorhinal cortex^52–54^. While men tend to outperform women in tasks of posterior hippocampus-dependent spatial navigation, women tend to outperform men in tasks of anterior hippocampus-, entorhinal cortex-, and perirhinal cortex-dependent face, landmark, and object recognition^54–62^. However, it remains unknown whether menstrual phase affects the sex differences reported for these tasks. If menstrual cycle effects on the hippocampus vary along its longitudinal axis, performance on tasks reliant on these different regions may also fluctuate in line with these brain alterations. In addition to menstrual phase, sex differences in spatial navigation may also depend on gender-related factors, including computer and videogame experience. Some studies show women in particular gain navigation performance benefits from videogame experience^63^, and others show no benefit of videogame experience on navigation performance^64,65^.

The current study asked whether sex differences in navigation depend on menstrual phase. If menstrual phase-dependent performance reflects previously reported underlying structural and functional changes in the hippocampus and entorhinal cortex, would a gender-related experiential factor such as videogame experience also influence performance? To address these questions, an online cross-sectional study of adults was conducted probing women and men on various core facets of medial temporal lobe-dependent navigation: cognitive map formation, topographical memory, face recognition memory, path integration, and self-reported navigation strategies. Due to sensitivity of the hippocampus to E2, comparisons were made between men, women in menstrual phases characterized by low-moderate E2 levels (EF + ML), and women in the menstrual phase characterized by the highest E2 levels (PO). Hypotheses are summarized below (Fig. 1B).

1. Posterior hippocampus-dependent topographical memory (adapted Four Mountains Task^66^), cognitive map formation (Cognitive Map Task^67^), and self-reported navigation strategy use (Navigational Strategies Questionnaire^16^): Aligning with presumed increased anterior hippocampus volume and concomitant decrease in posterior/anterior hippocampus volume ratio^24^, PO will show worse performance on these tasks and less self-reported cognitive map-based strategy use than EF + ML and men.
2. Anterior hippocampus- and entorhinal cortex-dependent face recognition (Cambridge Face Memory Test^68^): Aligning with presumed increased anterior hippocampus volume, PO will show better performance than EF + ML and men.
3. Entorhinal cortex-dependent path integration (Path and Location Integration Tasks^69^): If entorhinal cortex function is positively affected by E2^27^, PO will show better performance than EF + ML and men.
4. Exploratory analyses controlling for videogame experience were conducted to investigate whether a gendered factor, videogame experience, influenced sex- and menstrual cycle-dependent effects. If videogame experience improves spatial navigation ability in women, PO performance may particularly benefit.

## Results

### Demographic data

Four hundred twenty-three participants completed this online/virtual study. Of these, 240 participants were excluded for various eligibility reasons (Supplementary Table S1), leaving 183 participants.

Fifty-one percent of eligible cycling participants used an app or calendar to track their menstrual cycle. Of those who did not use an app or calendar (*n* = 63), all but three participants reported their estimations were “definitely” or “probably” accurate. Three participants reported one of their estimations “might not be accurate”, but no eligible participants reported their estimations were “definitely not accurate”. A total of 183 individuals (55 men and 128 women (including 42 EF, 46 PO, and 40 ML) were included in analyses for this study (Table 1). EF and ML groups were combined (EF + ML) to compare menstrual phases characterized by low-moderate E2 levels (EF and ML) to a menstrual phase characterized by highest E2 levels (PO).

**Table 1:** Demographic characteristics.

Characteristic	Total (*n* = 183; age range, 25–44)		Men (*n* = 55; age range, 28–44)		EF + ML women (*n* = 82; age range, 25–43)		PO women (*n* = 46; age range, 27–42)	
	Mean	SEM	Mean	SEM	Mean	SEM	Mean	SEM
Age (years)	34.37	0.34	33.13^b^	0.56	34.79	0.54	35.09	0.63
Education (years)	15.96	0.20	16.11	0.42	16.17	0.28	15.42	0.33
CES-D	16.22	0.92	17.87	1.75	15.74	1.42	15.09	1.64
GAD	5.09	0.36	5.64	0.70	4.87	0.54	4.85	0.63
Weekly video game experience (h)	5.31	0.52	8.24^a^	1.22	4.21	0.65	3.76	0.71
Menstrual cycle data								
Cycle length	–	–	–	–	28.35	0.23	28.05	0.37
Period length	–	–	–	–	3.98	0.15	4.96	0.23

*SEM* standard error of the mean, *PO* late follicular/periovulatory menstrual phase, *ML* mid/late luteal menstrual phase, *EF* early follicular menstrual phase, *CES-D* Center for Epidemiological Studies–Depression Scale, *GAD* General Anxiety Disorder-7 Scale. ^a^Significant (*p* < 0.05) post hoc Dunn’s test. Weekly video game experience: Men > EF + ML, PO. ^b^Trending (*p* < 0.10) post hoc t-test. Age: Men < EF + ML, PO.

Unless otherwise specified, normality (Quantile–Quantile plot) and homogeneity of variance (Residuals versus Fits Plot and Levene’s Test) assumptions were met for all analyses and parametric tests were used. Additional details regarding demographic data comparisons can be found in Supplementary Information “Demographic data”.

#### Demographic data: comparing men and women

Levene’s test showed the assumption of homogeneity of variances was violated for the age variable; therefore, the non-parametric Mann–Whitney *U* test was used to compare women (combining all menstrual phase groups) and men. Age differed significantly between women and men; women were older (*U* = 2690, *p* = 0.01). To control for potential age effects on navigation^70^, age was included as a covariate for models where sex was a grouping factor.

#### Demographic data: comparing men, EF + ML, and PO women

Parametric analysis of variance (ANOVA) showed there was a trend toward a significant effect of group on age (*F*(2,180) = 3.01, *p* = 0.052, *η*^2^ = 0.03); men were younger than EF + ML (*t*(180) = − 2.11, *p*_*Tukey*_ = 0.09, *d* = − 0.37) and PO (*t*(180) = − 2.17, *p*_*Tukey*_ = 0.08, *d* = − 0.43). There was no significant age difference between EF + ML and PO (*t*(180) = − 0.35, *p*_*Tukey*_ = 0.93, *d* = − 0.07). Due to trending age differences between groups, age was included as a covariate for models comparing men, EF + ML, and PO groups.

#### Videogame experience

Assumptions of homogeneity of variances and normality were violated for the comparison of videogame experience (“How many hours per week do you play video games?”) between women and men (combining all menstrual phase groups) and comparisons between men, EF + ML, and PO; therefore, Mann–Whitney *U* test was used to compare women and men, and Kruskal–Wallis test was used to compare men, EF + ML, and PO. There was a significant effect of sex on videogame experience; women reported fewer hours playing than men (*U* = 4756, *p* = 0.0001). There was also a significant effect of group on videogame experience when comparisons were made between men, EF + ML, and PO (*χ*^2^ = 14.48, *p* = 0.001); men reported significantly more hours playing than EF + ML (*z* = 3.42, *p* = 0.002) and PO (*z* = 3.21, *p* = 0.002). There were no significant differences in videogame experience between EF + ML and PO (*z* = 0.24, *p* = 0.81) (Table 1).

### Four Mountains and Cognitive Map Tasks (presumably posterior hippocampus-dependent)

All canonical result patterns are summarized in Fig. 1C. Seven participants did not complete the Four Mountains Task (3 EF, 1 ML, 2 PO, and 1 man). Additional quality control measures were used to exclude participants from Four Mountains Task analyses; three due to low quality data (2 men, 1 ML) and three more because they responded with the same button on every trial (1 EF, 1 ML, 1 man). Ten participants did not complete the Cognitive Map Task (3 PO, 3 ML, 4 men).

#### Sex effects: comparing women and men

Analyses of covariance (ANCOVAs) controlling for age were used to compare women and men on Four Mountains Task accuracy and number of trials to reach criterion on the Cognitive Map Task. There was not a significant main effect of sex on Four Mountains Task accuracy (*F*(1,167) = 0.16, *p* = 0.69, partial *η*^2^ = 0.001) or on number of trials to reach criterion on the Cognitive Map Task (*F*(1,170) = 0.17, *p* = 0.68, partial *η*^2^ = 0.001).

#### Menstrual phase effects: comparing men, EF + ML, and PO women

ANCOVAs controlling for age revealed there was not a significant main effect of group on Four Mountains Task accuracy (*F*(2,166) = 0.13, *p* = 0.88, partial *η*^2^ = 0.002) or number of trials to reach criterion on the Cognitive Map Task *F*(2,169) = 0.15, *p* = 0.86, partial *η*^2^ = 0.002).

### Navigational Strategies Questionnaire (presumably posterior hippocampus-dependent)

#### Sex effects: comparing women and men

**Figure 2:**
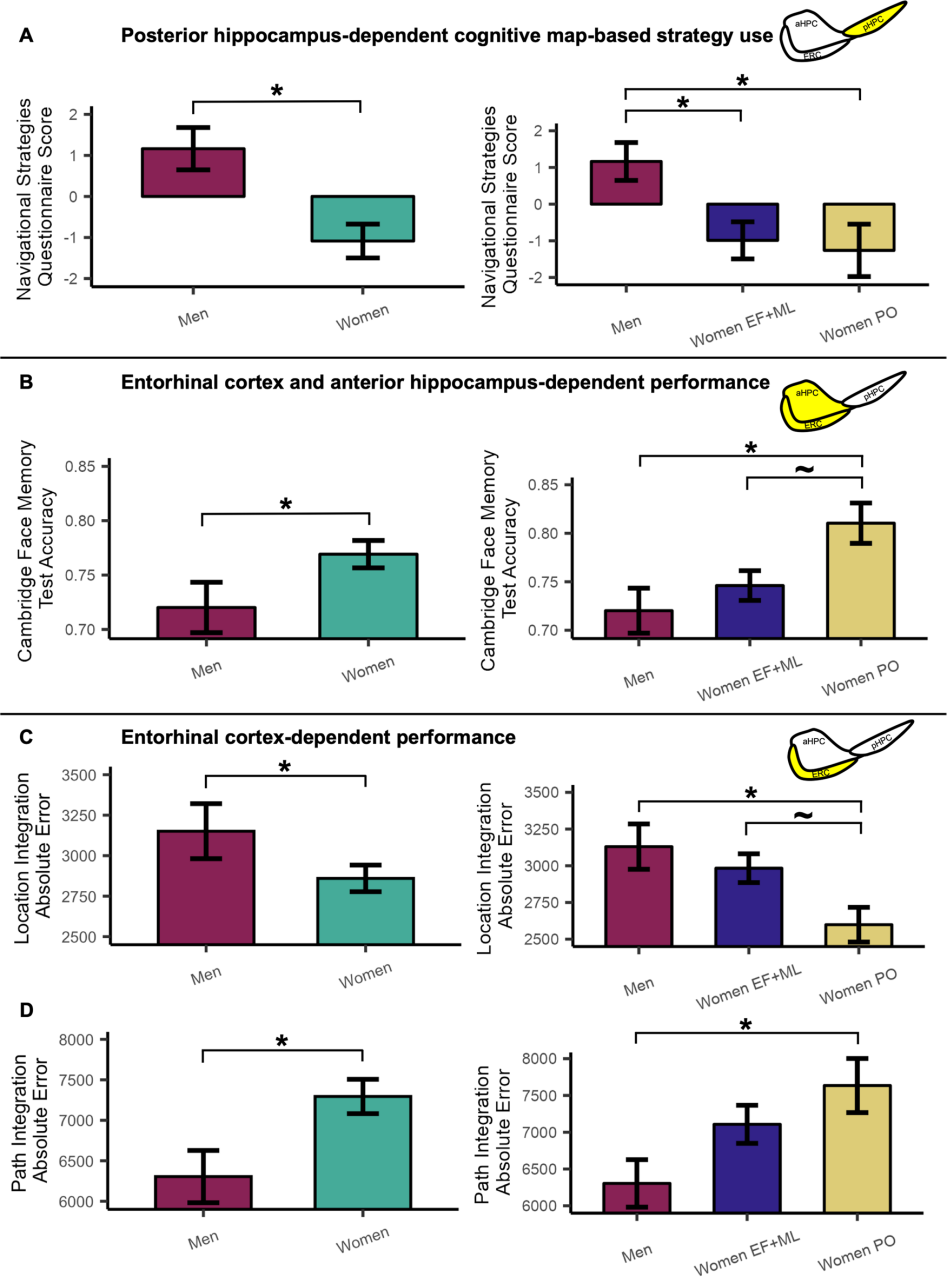
Barplots depicting sex and menstrual phase means and comparisons for task and questionnaire measures. (**A**) Navigation Strategies Questionnaire score; (**B**) Cambridge Face Memory Test accuracy (see Supplementary Fig. S1 for accuracy comparisons for Parts 1–3); (**C**) Location Integration Task absolute error; (**D**) Path Integration Task absolute error; (**D**) Yellow highlight = medial temporal lobe region that may be particularly relevant for the measure. *PO* late follicular/periovulatory menstrual phase, *ML* mid/late luteal menstrual phase, *EF* early follicular menstrual phase, *aHPC* anterior hippocampus, *pHPC* posterior hippocampus, *ERC* entorhinal cortex. *p < 0.05; ^~^*p* < 0.08.

ANCOVA controlling for age showed there was a significant main effect of sex on Navigational Strategies Questionnaire scores (*F*(1,180) = 10.78, *p* = 0.001, partial *η*^2^ = 0.06); compared to women, men demonstrated increased tendency to report use of posterior hippocampus-dependent map-based navigation strategies. Compared to men, women demonstrated increased tendency to use scene-based strategies (Fig. 2A).

#### Menstrual phase effects: comparing men, EF + ML, and PO women

ANCOVA controlling for age showed there was a significant main effect of group on scores for the Navigational Strategies Questionnaire (*F*(2,179) = 5.43, *p* = 0.005, partial *η*^2^ = 0.06); post hoc comparisons showed men reported significantly increased use of map-based strategies compared to PO (*t*(179) = 2.86, *p*_*Tukey*_ = 0.01, *d* = 0.58) and EF + ML (*t*(179) = 2.91, *p*_*Tukey*_ = 0.01, *d* = 0.51), who reported increased use of scene-based strategies. For Navigational Strategies Questionnaire scores, there was not a significant difference between EF + ML and PO (*t*(179) = 0.36, *p*_*Tukey*_ = 0.93, *d* = 0.07) (Fig. 2A).

### Cambridge Face Memory Test (presumably anterior hippocampus- and entorhinal cortex-dependent)

All eligible participants passed the data quality control measures assessed for the Cambridge Face Memory Test. During the simplest sensory/part-based component of the task (recognizing the studied faces from the same angles (e.g., full profile) and lighting conditions; Fig. 4B, Part 1), Q-Q plots were skewed for comparisons of women and men and comparisons of men, EF + ML, and PO; therefore, Part 1 accuracy data was winsorized.

#### Sex effects: comparing women and men

ANCOVA controlling for age was used to compare women and men on overall accuracy. Women performed significantly better than men on overall accuracy (*F*(1,180) = 4.00, *p* = 0.047, partial *η*^2^ = 0.02) (Fig. 2B).

Exploratory analyses were conducted to assess which task parts were driving the significant sex difference. ANCOVA controlling for age revealed there was not a significant main effect of sex on accuracy during Part 1 (*F*(1,180) = 0.007, *p* = 0.93, partial *η*^2^ = 0.00004). For the more challenging holistic processing-dependent component of the task (recognizing the studied face from different views and/or lighting conditions; Fig. 4B, Part 2), ANCOVA controlling for age revealed women performed significantly better than men (*F*(1,180) = 3.99, *p* = 0.047, partial *η*^2^ = 0.02). During the most difficult part of the task (recognizing the studied face from a different view with overlaid visual noise), thought to be most dependent on holistic processing and the entorhinal cortex^54^, ANCOVA controlling for age revealed there was not a significant main effect of sex on accuracy (*F*(1,180) = 2.19, *p* = 0.14, partial *η*^2^ = 0.01; Fig. 4B, Part 3).

#### Menstrual phase effects: comparing men, EF + ML, and PO women

ANCOVA controlling for age revealed a significant main effect of group on overall face recognition accuracy (*F*(2,179) = 4.75, *p* = 0.01, partial *η*^2^ = 0.05). Post hoc comparisons showed PO outperformed men (*t*(179) = − 3.00, *p*_*Tukey*_ = 0.01, *d* = − 0.61). There was a trend toward PO outperforming EF + ML (*t*(179) = − 2.32, *p*_*Tukey*_ = 0.055, *d* = − 0.43). There were no overall accuracy differences between EF + ML and men (*t*(179) = − 1.02, *p*_*Tukey*_ = 0.57, *d* = − 0.18) (Fig. 2B).

Exploratory analyses were conducted to assess which parts of the task were driving significant group differences. ANCOVA controlling for age showed there was not a significant main effect of group on Part 1 accuracy (*F*(2,179) = 1.06, *p* = 0.35, *η*^2^ = 0.01). ANCOVA controlling for age revealed there was a significant main effect of group on Part 2 accuracy (*F*(2,179) = 3.76, *p* = 0.03, partial *η*^2^ = 0.04). Post hoc comparisons showed PO outperformed men (*t*(179) = − 2.72, *p*_*Tukey*_ = 0.02, *d* = − 0.55). For Part 2 accuracy, there were no significant differences between EF + ML and PO (*t*(179) = − 1.86, *p*_*Tukey*_ = 0.15, *d* = − 0.34) and EF + ML and men *t*(179) = − 1.18, *p*_*Tukey*_ = 0.47, *d* = − 0.21). ANCOVA controlling for age also showed a significant main effect of group on Part 3 accuracy (*F*(2,179) = 4.26, *p* = 0.016, partial *η*^2^ = 0.05). Post hoc comparisons showed PO significantly outperformed EF + ML (*t*(179) = − 2.50, *p*_*Tukey*_ = 0.035, *d* = − 0.46) and men (*t*(179) = − 2.69, *p*_*Tukey*_ = 0.02, *d* = − 0.54). For Part 3 accuracy, there were no significant differences between EF + ML and men (*t*(179) = − 0.47, *p*_*Tukey*_ = 0.89, *d* = − 0.08). See Supplementary Fig. S1 for Parts 1–3 results summary.

### Location and Path Integration Tasks (presumably entorhinal cortex-dependent)

Two participants did not complete the Location and Path Integration tasks (1 PO woman, 1 man). Levene’s test showed the assumption of homogeneity of variances was violated for Location Integration Task absolute error when making comparisons between women and men and between EF + ML, PO, and men; therefore, Location Integration Task absolute error was winsorized.

#### Sex effects: comparing women and men

For the Location Integration Task, ANCOVA controlling for age revealed a significant main effect of sex; women made significantly smaller errors than men (*F*(1,178) = 4.30, *p* = 0.04, partial *η*^2^ = 0.02) (Fig. 2C).

For the Path Integration Task, ANCOVA controlling for age showed a significant main effect of sex; women made larger errors than men (*F*(1,178) = 4.77, *p* = 0.03, partial *η*^2^ = 0.03) (Fig. 2D).

### Menstrual phase effects: comparing men, EF + ML, and PO women

For the Location Integration Task, ANCOVA controlling for age showed there was a significant main effect of group on absolute error (*F*(2,177) = 4.13, *p* = 0.018, partial *η*^2^ = 0.04); post hoc comparisons showed PO made smaller errors than men (*t*(177) = 2.80, *p*_*Tukey*_ = 0.016, *d* = 0.57) and trended toward making smaller errors than EF + ML (*t*(177) = 2.19, *p*_*Tukey*_ = 0.076, *d* = 0.41). There were no significant error differences between EF + ML and men (*t*(177) = 0.92, *p*_*Tukey*_ = 0.63, *d* = 0.16) (Fig. 2C).

For the Path Integration Task, ANCOVA controlling for age showed there was a significant main effect of group on absolute error (*F*(2,177) = 3.07, *p* = 0.049, partial *η*^2^ = 0.03); post hoc comparisons showed PO made larger errors than men (*t*(177) = -2.46, *p*_*Tukey*_ = 0.04, *d* = − 0.50). There were no significant error differences between EF + ML and PO (*t*(177) = − 1.17, *p*_*Tukey*_ = 0.47, *d* = − 0.22) and EF + ML and men (*t*(177) = − 1.60, *p*_*Tukey*_ = 0.25, *d* = − 0.28) (Fig. 2D).

#### Correlating Location Integration Task and Cambridge Face Memory Test performance

Given that face recognition and location integration abilities fluctuated similarly across the menstrual cycle, exploratory correlational analyses were conducted to estimate whether there may be a common factor underlying these abilities (e.g., holistic processing^55,69^).

**Figure 3:**
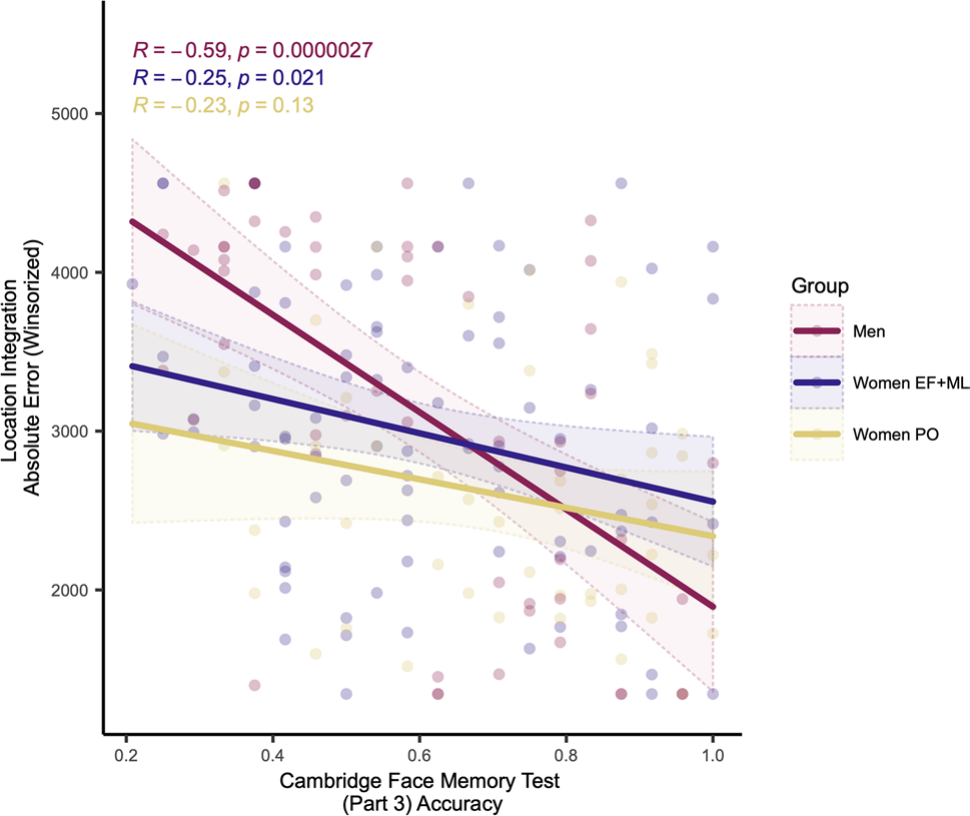
Scatter plot depicting exploratory correlation relationships between Cambridge Face Memory Test (Part 3) accuracy and Location Integration Task absolute error by sex and menstrual phase. *PO* late follicular/periovulatory menstrual phase, *ML* mid/late luteal menstrual phase, *EF* early follicular menstrual phase. Plotted with 95% confidence intervals.

EF + ML showed a significant small negative relationship between Location Integration absolute error and performance on Part 3 of the Cambridge Face Memory Test (*r*(80) = − 0.25, *p* = 0.02). Men showed a significant large relationship between Location Integration absolute error and Cambridge Face Memory Test performance (*r*(52) = − 0.59, *p* = 0.000002). PO showed no significant relationship (*r*(43) = − 0.23, *p* = 0.12) (Fig. 3).

### Exploratory analyses: videogame experience

Additional exploratory analyses controlling for videogame experience (self-reported number of hours playing videogames per week) were conducted to investigate whether sex- and menstrual cycle-dependent effects were maintained above and beyond variation in videogame experience. Full results are reported in Supplementary Information (‘Results Controlling for Videogame Experience’). Results including the videogame experience covariate were largely consistent with results when experience was omitted with two exceptions; when including the videogame experience covariate, significant main effects of sex disappear for Location and Path Integration Tasks and a significant main effect of group (comparing Men, EF + ML, and PO) disappears for the Path Integration Task.

## Discussion

This study emphasizes a dynamic sex- and menstrual cycle-influenced model of spatial navigation performance and strategy by comparing the relative influences of sex and menstrual phase on multiple facets of medial temporal lobe-dependent cognition: (1) posterior hippocampus-dependent cognitive map formation, topographical memory, and self-reported navigation strategy use, (2) anterior hippocampus- and entorhinal cortex-dependent face recognition memory, and (3) entorhinal cortex-dependent path integration.

### Sex (but not menstrual phase) influences some aspects of cognition presumed to be mediated by posterior hippocampus

Contrary to hypotheses, there were no sex or menstrual phase performance differences on posterior hippocampus-dependent tasks, consistent with past literature showing that posterior hippocampus volume may not increase during PO^24^. A lack of sex difference on Four Mountains Task performance, which relies in part on mental rotation, aligns with research showing sex differences disappear when mental rotation becomes more like manual rotation (e.g., when rotating three-dimensional photographs rather than two-dimensional line drawings)^71^. Contrary to previous findings demonstrating an advantage for men in forming/using cognitive maps, there were no sex or menstrual phase differences in Cognitive Map Task performance^39,72^. This discrepancy may be due to differences in age groups studied (e.g., the current study: age 25–44 years; Burles et al.^72^: mean age ~ 20 years; Liu et al.^39^: 18–67 years). Cognitive map formation depends on a variety of cognitive processes, including path integration, which is thought to aid in setting the scale of cognitive maps^67,73^. Given the presence of landmarks in the Cognitive Map Task, the observed lack of sex and menstrual phase differences in performance may be due to increased path integration ability (when landmarks are absent) in men and location integration (when landmarks are present) in PO, leading to no overall difference.

As hypothesized, compared to women, men reported using more posterior hippocampus-dependent cognitive map-based strategies. This sex difference is important given that greater reported cognitive map usage has been correlated with better navigation performance^16^. Both groups of women (PO and EF + ML) were more likely to report use of anterior hippocampus-dependent scene-based strategies than men. Thus, self-reported map-based strategy use may not be menstrual phase-dependent. It is important for future research to consider that, compared to task performance, self-reported navigation ability and strategy may be less sensitive to steroid hormone cycling.

Previous studies using the original Four Mountains Task have demonstrated positive correlations between task performance and both posterior and anterior hippocampus volume^74^. Given past work showing anterior hippocampus volume increases during PO^24^, it is interesting to consider that success on the Four Mountains Task may depend on the anterior hippocampus during PO and the posterior hippocampus during EF and ML. Future studies should consider whether volume fluctuations along the longitudinal hippocampus axis across the menstrual cycle affect navigation.

### Menstrual phase influences cognition presumed to be mediated by anterior hippocampus and entorhinal cortex

This study is among the first to examine predictions about face recognition ability across the menstrual cycle (without the use of emotional faces^75^). PO had better face recognition than men, particularly when recognition was likely most dependent on holistic processing^54,55,76^. These results are consistent with past research showing women are better at recognizing faces than men, and suggest previously reported sex differences in face recognition may be driven by the difference between PO and men^77,78^.

In this study, PO performed less well than men on the entorhinal cortex-dependent Path Integration Task. However, EF + ML performed similarly to men, providing the first evidence that sex differences in path integration may be modulated by menstrual phase. Men tend to outperform women on navigation tasks in environments devoid of landmarks^65^, possibly due to improved visuospatial working memory^79^. Because of the landmark absence during encoding, this Path Integration Task depends heavily on visuospatial integrative processes. Thus, the current study suggests the presence or absence of a landmark could explain previous variability of findings in the few extant studies showing no sex differences in navigation ability^43,72,80^. The neuronal circuits for strong path integration performance exist in both women and men, but in women their activation may be contingent on menstrual phase.

In contrast to Path Integration Task results and consistent with the proposed importance of landmarks, PO outperformed EF + ML and men on the Location Integration Task. Although this task is similar to the Path Integration Task, a target location was depicted *with a landmark* during encoding, allowing participants to encode the target location using intra-scene visuospatial processes^81^. PO’s better performance on the Location Integration Task is consistent with past work showing women perform better when salient landmark information is available^41^. If high E2 facilitates improved performance on tasks involving integrative/holistic processing (e.g., Cambridge Face Memory Test and Location Integration Task), the anterior hippocampus, previously shown to increase in volume during PO^24^, may be critical for supporting this processing.

This study is the first to suggest the PO phase is associated with increased anterolateral entorhinal cortex-dependent face memory and Location Integration Task performance and decreased posteromedial entorhinal cortex-dependent Path Integration Task performance. Entorhinal cortex volume negatively correlates with progesterone levels and is lowest during the ML phase^82^. The current study did not show significant performance differences between EF and ML phases (Supplementary Information ‘All menstrual phase effects: results comparing men, EF, PO, and ML women’), suggesting the relationship between progesterone and entorhinal cortex-dependent cognition is not simple, especially given that entorhinal cortex function may differ along its axes. The anterolateral entorhinal cortex is thought to underlie within-object configural processing and travelling between landmarks, while posteromedial entorhinal cortex underlies scene processing and travelling between locations (without landmarks) with path integration cues alone^83,84^. It is possible that E2 and progesterone effects on entorhinal cortex volume are subregion-specific. Future work should address whether high E2 and progesterone levels negatively influence the posteromedial entorhinal cortex but not the anterolateral entorhinal cortex.

Path Integration Task error was the only behavioural outcome affected by statistically acknowledging videogame experience (Supplementary Information ‘Results controlling for videogame experience) in terms of loss of significant main effects of sex and menstrual phase grouping. These results are consistent with past work showing videogame experience boosts navigation performance for women particularly in the absence of cues/landmarks^63^. Future work should probe interactive influences of menstrual phase and videogame experience on navigation.

### Does E2 support holistic/integrative processing?

Location Integration Task and face recognition performance fluctuated similarly across the menstrual cycle. Both EF + ML women and men (but not PO women) showed significant correlations between face recognition and Location Integration abilities, such that better face recognition during the most holistic processing-dependent face memory trials was associated with decreased Location Integration Task error. Understanding of why performance on these tasks is similar when E2 and progesterone levels are likely low is lacking.

E2 may modulate visual processing and memory abilities more broadly. Given that decreased E2 levels after ovariectomy in rodents are associated with reduced dendritic spine density on pyramidal neurons in the hippocampal cornu ammonis 1 subfield^85^, and E2 administered systemically or directly into the hippocampus increases dendritic spine density and enhances performance on a variety of cognitive tasks^86^, E2 may modulate integration functions of the hippocampus based on visible features such as landmarks or face parts, contributing to performance on both face recognition and Location Integration tasks^55,87^.

It is important to acknowledge the large functional neural networks beyond the hippocampus underlying navigation. For example, survey knowledge depends on various structures, including the retrosplenial cortex and parieto-occipital sulcus^11–14^. These neural networks may also be influenced by the menstrual cycle. Previous transcranial magnetic stimulation studies have demonstrated high intracortical excitability in visual and somatosensory cortex in women when E2 levels are high^88^. A within-subject dense-sampling study of one naturally-cycling woman showed positive relationships between E2 levels and resting-state nodal association strength within Visual, Default Mode, and Dorsal Attention Networks^89^. Enhanced functional connectivity between critical nodes of a network such as the Dorsal Attention Network in the presence of high E2 could drive an increase in a broad range of perceptual sensitivities to various stimuli types. Thus, it is possible that better face recognition and Location Integration Task performance during PO is influenced by high E2 leading to greater involvement of the hippocampus in integrative aspects of cognition and/or enhanced general visual processing abilities^55,87^.

### Are there differences between navigation and memory?

The current study may speak to important differences between navigation and memory. Successful navigation depends on knowing which forms of memory and sensory information (idiothetic vs. visual) are most important. Past work suggests certain tasks, like path integration, place less demand on memory, while others, like recognizing facial features, place greater demand on memory^90^. In the current study, if men were better able to use *nonvisual distance cues* to calibrate their path integration systems compared to women^91^, memory-related *visual cues* may have been particularly beneficial for path integration processes in women. These visual cues may have been especially important during the PO phase when women were less proficient at using nonvisual distance cues. Thus, high E2 during PO could particularly benefit tasks that place greater demand on memory.

This work emphasizes the importance of hormone variation when assessing sex differences in spatial navigation performance, in particular presumed variation in E2 level. To best understand how progesterone levels influence path integration and face recognition abilities presumably mediated by entorhinal cortex, future studies should capture more fine-grained windows of progesterone fluctuation by comparing performance on days 3–5 of EF and 7–10 days prior to menses during ML. Since E2 and progesterone were not measured directly, their unique effects on navigation could not be precisely determined. Despite these limitations, this study showed systematic performance variations across the menstrual cycle, laying groundwork for future studies correlating performance with hormone levels that may better address questions regarding distinct hormonal effects. Our study overcame some of the limitations of previous work investigating the effects of sex and menstrual phase on only one navigation-related skill by assessing multiple navigation domains, improving understanding of the mechanisms underlying potential effects of the menstrual cycle on navigation and better capturing the range of cognitive processes involved in real-world navigation.

A potential study limitation is that online testing was used instead of in-person testing. Nevertheless, various attention checks were distributed throughout the survey and data quality was carefully assessed. Online testing also allowed for recruitment of large numbers not typically obtainable in studies of the menstrual cycle and cognition. Even though it was not feasible to recruitment to planned pre-registered final cohort size goals due to participant eligibility constraints, effect sizes in this study ranged from small to medium in size, which is typical for studies of sex differences in human spatial navigation^38^. Additionally, effect sizes for comparisons of all women to men tended to be small, while effect sizes for comparisons of PO to men were typically medium, reinforcing the idea that it is crucial to consider menstrual phase when investigating cognitive sex differences.

Reliance on self-report for menstrual phase characterization is not ideal. Nonetheless, although we did not assay hormones directly, there is evidence that self-report data used to determine participants’ cycle phase is well-aligned with serum hormone levels, suggesting self-reported menstrual phase may appropriately reflect hormone fluctuation^49^. Including only women who used a calendar or period-tracking app to track their menstrual phase or who reported high confidence in their answers allowed for good approximation of cycle day and length gave some assurance that phase assumptions were accurate. Future work employing within-subjects designs will be needed to confirm the current study’s findings and ensure results were not due to sampling differences between groups. When using cross-sectional designs, assay confirmation of hormonal status will be critical for future research to make conclusions based on hormone level effects. Historically, research has not adopted consistent methods for operationalizing the menstrual cycle and more integrative guidelines and standardized tools for studying the menstrual cycle as an independent variable should be used in future work^92^.

Although hypotheses were based on consideration of steroid hormone effects on neural circuitry, the current study did not directly assess the underlying neural circuitry alterations that could be influencing behaviour. Further, it is important to consider that variation in spatial performance and its underlying neural circuitry may be related to fluctuation of additional hormones, such as testosterone. For example, transgender men receiving gender-affirming testosterone-based hormone therapy perform as well as cisgender men on mental rotation tasks^93^. In women, higher testosterone levels have been associated with improved navigation performance and increased medial temporal lobe activity during successful navigation^94,95^. Future studies should assess the functional neuroanatomy of navigation across different steroid hormone concentrations and treatments.

## Conclusions

Results from this study point to the importance of taking menstrual phase into account to explain sex differences in navigation performance and strategy. Fluctuation of steroid hormone levels across the menstrual cycle may alter an extensive range of perceptual sensitivities, including those involved with complex visual processing that affect performance in some aspects of navigation, such as memory processes involving objects/landmarks, but not others, such as processing of metric distances in the absence of landmarks.

The lack of attention paid to menstrual cycle effects in all research has resulted in deficient understanding of endocrine effects on cognition. In addition to menstrual cycling, other relevant considerations may include hormonal contraceptives, hormone therapy, ovarian removal, and conditions known to alter hormone levels, such as polycystic ovarian syndrome. This study demonstrated clear variation in performance on spatial navigation tasks across the menstrual cycle as predicted by hormonal effects on neural structures mediating task performance. This endocrine-informed perspective will enhance rigor and expand the relevance of neuropsychological research.

## Methods

### Participants

A total of 183 individuals (55 men and 128 women) were included in analyses for this online/virtual study (Table 1). The target cohort size was 200 participants, with an original aim to recruit 263 participants, assuming approximately 20–30% of recruited participants would not meet eligibility criteria. Given 56.74% of recruited participants were not eligible for inclusion in final analyses, it was not feasible to continue recruitment to reach pre-registered final cohort size goals.

Questions, methods, and hypotheses were preregistered for this study. All data collected for this study, along with our analyses, design, and procedure can be accessed on the Open Science Framework at https://osf.io/nakd5. Amazon’s Mechanical Turk (MTurk; mturk.com) was used for online recruitment of individuals assigned males at birth or assigned females at birth self-identifying as men or women, respectively. Inclusion criteria were English fluency, age between 25 and 45 years, and regular menstrual cycles defined as consistent menstrual periods every 21–35 days within six months prior to completing the study for women^96^. Exclusion criteria were: for women, current pregnancy, breastfeeding within six months prior to completing the study, perimenopause/menopause, and for all participants, history of disorders or conditions known to alter hormone levels (e.g., polycystic ovarian syndrome), dementia, stroke, and/or hormone therapy/contraceptive use.

### Menstrual phase

Menstrual phase was determined by self-report via questions administered online (Supplementary Information ‘Menstrual cycle phase categorization’). Given that it is not appropriate to ‘standardize’ cycles to a 28-day norm, the luteal phase was assumed to be 14 days long (i.e., the relatively robust length of the luteal phase, irrespective of overall cycle length^97^), while the follicular phase was assumed to vary in length. Cycle day was calculated by subtracting the start date of the participant’s last menstrual period from the day they completed the study. Since a 28-day cycle cannot always be assumed and cycles vary from woman to woman, participants were asked to state the typical length of their cycle^96^. As an online study, absolute hormone levels were not measured, and these groupings were approximations (Fig. 1A). When care is taken, estimates of hormone variation based on self-report are well-aligned with objective hormone levels^49^. Because menstrual phase was self-reported, participants were asked to indicate the confidence they had in their estimations (options included: definitely accurate, probably accurate, might not be accurate, or definitely not accurate). Participants were excluded if they indicated any of their answers were “definitely not accurate”.

Women were categorized as being in the early follicular phase (EF) if they self-reported their current cycle day to be in the first half of the follicular phase. For example, a participant who was on cycle day nine and reported a 34-day long cycle was categorized as EF, assuming their follicular phase was lengthened compared to someone with a 28-day cycle. Although hormones were not directly measured, EF tends to be characterized by low circulating levels of E2 and progesterone.

Women were categorized as being in the late follicular/periovulatory phase (PO) if they self-reported their current cycle day to be in the latter half of the remaining days leading up to the periovulatory phase. For example, a participant who was on cycle day 12 of a 32-day long cycle was categorized as PO, again assuming their follicular phase was lengthened compared to someone with a 28-day cycle. Although hormones were not directly measured, PO tends to be characterized by rising/high circulating levels of E2 and low levels of progesterone.

Women were categorized as being in the mid/late luteal phase (ML) if they self-reported their current cycle day to be 1–11 days prior to menses onset. For example, a participant who was on cycle day 22 of a 30-day long cycle was categorized as ML because their current cycle day was eight days prior to their estimated menses onset. Although hormones were not directly measured, ML tends to be characterized by moderate circulating levels of E2 and high levels of progesterone. Due to recruitment constraints and to better address hypotheses dependent on low-moderate E2 levels compared to high E2 levels, EF and ML groups were combined (EF + ML) and compared to PO and men (contrary to preregistration plans to separately compare EF, PO, ML, and men). Results for preregistered planned comparisons of all groups can be found in Supplementary Information (‘All menstrual phase effects: results comparing men, EF, PO, and ML women').

### Experimental design and procedure

This experiment was administered virtually/online via Qualtrics (Qualtrics, Provo, UT). Participants first completed The Navigational Strategies Questionnaire to test the hypothesis that self-reported posterior-hippocampus dependent cognitive map-based strategy use and navigation performance will depend on menstrual phase. The Navigational Strategies Questionnaire categorizes individuals as either “scene-based” or “cognitive map-based” navigators^16^. They also answered a demographic questionnaire, as well as the Center for Epidemiological Studies-Depression scale and Generalized Anxiety Disorder questionnaire to ensure groups were well-matched on measures of depressive mood and anxiety.

Next, participants were directed to a separate browser, where they completed four cognitive tasks (gettinglost.ca). Given that orienting within an environment is a complex skill relying on several cognitive functions, tasks that assessed several orientation skills independently, as well as other cognitive functions, such as recognition memory, were administered. This battery included tasks of posterior hippocampus-dependent topographical memory (Four Mountains Task^66,98^) and cognitive map formation (Cognitive Map Task^67^), anterior hippocampus- and entorhinal cortex-dependent face recognition memory (Cambridge Face Memory Test^68^), and entorhinal cortex-dependent path integration with and without a target object (Location and Path Integration Tasks, respectively^69^). Tasks were administered in the following order: Four Mountains Task, Cambridge Face Memory Test, Path Integration Task, Location Integration Task, and Cognitive Map Task. Participants had 24 h to complete the online study and were instructed to complete the session in a distraction-free, quiet/private room on a computer at a desk or table. Upon completing the study, participants were compensated $7.00 USD.

### Tasks

#### Four Mountains Task

**Figure 4:**
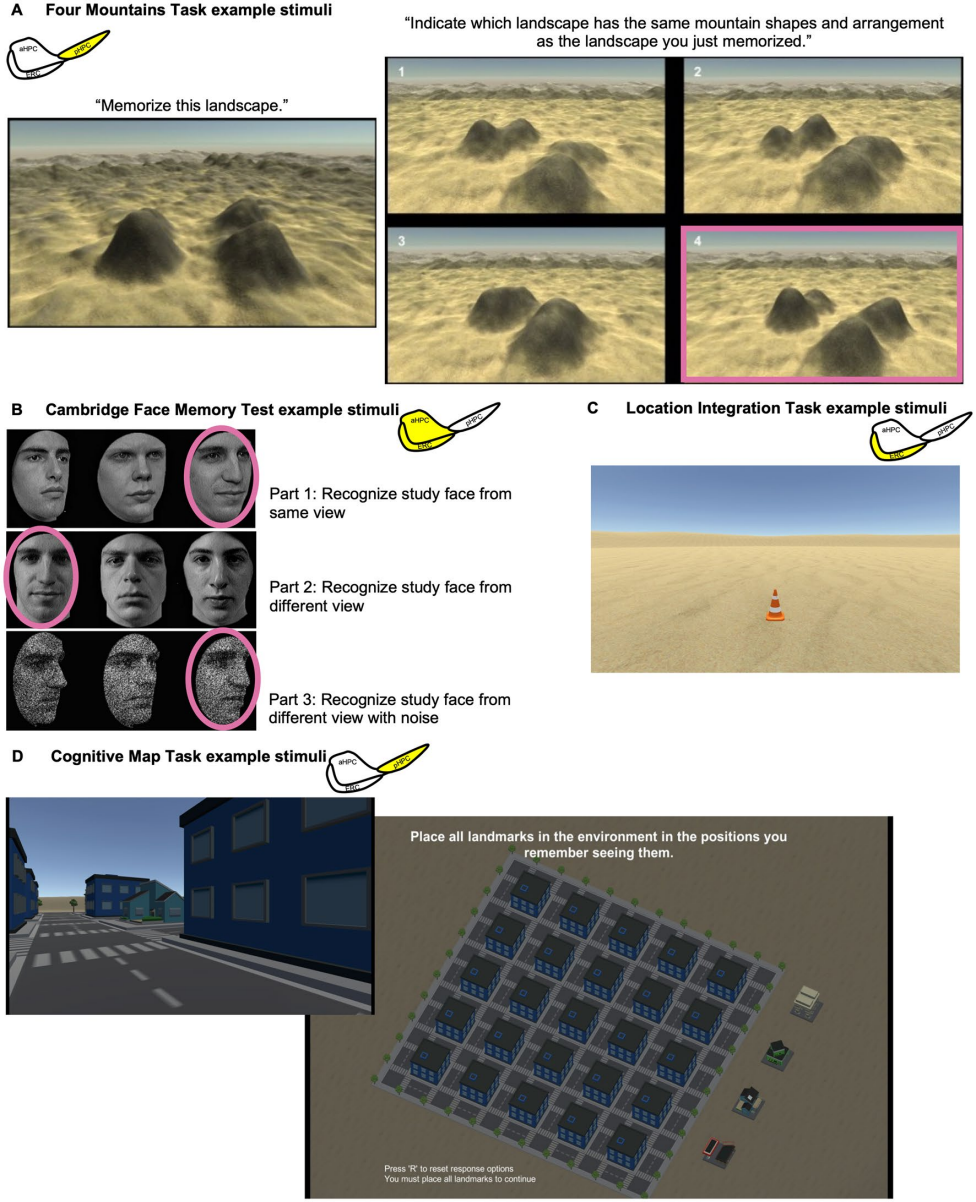
Graphic depicting example stimuli for cognitive tasks, including the Four Mountains Task, Cambridge Face Memory Test, and Cognitive Map Task example stimuli. (**A**) Four Mountains Task example stimuli for encoding and recognition trials with pink square denoting correct answer; (**B**) Cambridge Face Memory Test example stimuli for Parts 1–3 with circles around the faces denoting correct answers (image reproduced from Duchaine and Nakayama^68^ with permission from authors); (**C**) Location Integration task example landscape with orange traffic cone stimuli; (**D**) Cognitive Map Task example stimuli including encoding and testing phases. Yellow highlight = medial temporal lobe region that may be particularly relevant for the measure.

The GettingLost.ca Four Mountains Task was designed to test the hypothesis that posterior hippocampus-dependent spatial performance depends on menstrual phase. For each of the 20 trials of the Four Mountains Task (Fig. 4A), participants studied a landscape populated with four protuberances representing mountains in the foreground. They had eight seconds to memorize this scene and were instructed to focus on the shape and arrangement of the mountains in the foreground. After a two second delay, the stimulus was followed by four similar landscapes seen from different viewpoints and under different conditions of lighting or weather. One of the four pictures showed the same place as in the previous picture, although it was depicted from a slightly different viewpoint with different environmental conditions. The participant’s task was to identify which of the four pictures showed the same place as the one they had just seen. Response options remained until a response was provided. The dependent variable of interest was accuracy (number of correct trials divided by total number of trials).

#### Cambridge Face Memory Test

The Cambridge Face Memory Test is a face recognition task that was administered to test the hypothesis that entorhinal cortex- and anterior hippocampus-dependent memory performance depends on menstrual phase. This task involved first studying faces with the presentation of an individual face from three angles, with three test parts: (1) recognizing the studied face from the same angle, (2) recognizing the studied face from a different angle, and (3) recognizing the studied face from a different angle with visual noise (Fig. 4B image reproduced from Duchaine & Nakayama with permission from authors^68^).

To begin the task, three study images were presented for three seconds each. The images were a left 1/3 profile, a frontal view, and a right 1/3 profile. In Part 1, three test items were then presented (top panel, Fig. 4B), and participants were instructed to pick the individual whom they were just studying. Each test item was identical to the study item (viewed from the same angle with the same lighting). Part 1 could be accomplished using sensory or part-based methods dependent on intact processing of individual face features^54^. There were six target faces, and this procedure was repeated for the five remaining target faces. Target faces were never used as distracter faces. A single review image that had a frontal shot of each target face was then presented, and participants were given 20 s to review this image.

In Part 2, participants were presented with 30 forced choice test items (6 target faces × 5 presentations) in a fixed, random order. Each test item contained three faces, one of which was a target face. They were instructed that each test item would contain one of the six target faces and told to respond with the key corresponding to the number under the target face. All were novel images in which the lighting, pose, or both varied (middle panel, Fig. 4B). Part 2 could be accomplished using part-based processing or higher-level holistic processing^54,76^. Participants were presented with the review image again for 20 s.

In Part 3, 24 test items (6 target faces × 4 presentations) were presented in a fixed, random order. These items consist of novel images depicting a varied pose with different levels of Gaussian noise overlaid (bottom panel, Fig. 4B). Levels of noise for the faces in a test item were identical. Part 3 could be accomplished using holistic processing, which facilitates creation of robust face representations that can be used for recognition across changes in viewpoint and expression^54,76^. The dependent variables of interest included overall task accuracy, as well as accuracy for each of the three test parts.

#### Path Integration Task

The Path Integration Task assesses the ability to integrate optic flow into a coherent sense of movement, and update and store that representation for future use. This task was administered to test the hypothesis that entorhinal cortex-dependent spatial performance depends on menstrual phase. For each of the 18 trials of the Path Integration Task, participants were placed in an empty desert-like environment, and the camera would automatically move forward, make a turn, then move forward again, forming two arms of a triangle. After the automatic movement, participants used the left and right arrows (or the “a” and “d” keys) to face the position from which the automatic movement was started. Once they were finished turning, they pressed “Enter” and could then move forwards using the up arrow (or “w” key) and try to stop on the same place where the trial began, completing the third arm and ‘closing the triangle’. Again, when the participants were finished moving, they pressed “Enter”. Performance on each trial was quantified as the average absolute error (i.e., the straight-line distance from the ending location to the starting location of that trial).

#### Location Integration Task

The Location Integration Task similarly assesses the ability to integrate optic flow into a coherent sense of movement, but has participants remember the location of an object (traffic cone), instead of integrating an outbound path from movement alone (as is the case for the Path Integration Task). This task was administered to test the hypothesis that entorhinal cortex-dependent spatial performance will depend on menstrual phase.

For each of the 12 trials of the Location Integration Task, a bare desert-like landscape with an object (orange traffic cone) in the distance was presented (Fig. 4C). The camera would automatically turn and face the cone, pause, and then return to its starting orientation. The screen would then fade to black, and the traffic cone would disappear. The screen would then fade back in, and participants were asked to use the left and right arrows (or the “a” and “d” keys) to face the position where they saw the cone. Once they were finished turning, participants pressed “Enter”. They were then asked to move to the location they last saw the cone using the up arrow (or “w” key). Again, when they were finished moving, they would press “Enter” to conclude their response. Performance on each trial of this task was quantified as the average absolute error (i.e., the straight-line distance from their ending location to the location where the cone was last seen).

#### Cognitive Map Task

The Cognitive Map Task assesses an individual's ability to acquire survey knowledge and generate a mental representation of a simple, city-like virtual environment (Fig. 4D). This task was administered to test the hypothesis that posterior hippocampus-dependent spatial performance will depend on menstrual phase.

The environment includes four distinct landmarks embedded in a 5-by-5 grid of identical buildings. For each trial of this task, participants visited each distinct landmark during a passive, first-person movement through the same environment. At the end of each trial, an aerial view of the environment was presented and participants were asked to place icons for each landmark in their corresponding locations. The drag-and-drop response screen remained until the response was complete. The task ended when all four landmark icons were placed correctly or after an upper limit of 12 trials. The virtual city remained the same across trials, but the automatic path taken from trial to trial would change. If the correct environmental layout was not provided after the 12th trial, the task ended. The dependent variable of interest for this task was the number of trials needed to successfully place all four landmark icons correctly.

### Data quality

Data quality control measures were used to exclude participants completing the Four Mountains Task and Cambridge Face Memory Test. A multivariate model was used to identify accuracy and performance outliers at the participant level, with the specific aim to identify participants responding without attempting to perform the task. A quality score was used to estimate how unlikely a participant’s accuracy and reaction time were compared to the whole cohort. This was used to identify participants with low accuracy and atypically fast reaction times, which was interpreted as indicating they were guessing without effort (Supplementary Information ‘Data quality assessment’). Additionally, participants were excluded from the Four Mountains Task analyses if they were flagged for responding with the same physical button at every trial.

### Statistical analysis

All analyses were performed using R Statistical Software (v4.2.0; R Core Team 2022). Model assumptions of homogeneity of variances and normality were evaluated using Levene’s test, Residuals versus Fits Plots, Quantile–Quantile (Q–Q) plots, and skewness assessment. The goal of the first set of analyses was to assess sex differences in task and questionnaire scores. Women in all menstrual phases were combined and compared to men using one-way analyses of covariance (ANCOVAs), with sex as a between-subjects factor (two levels including men and women), controlling for age.

The goal of the second set of analyses was to assess whether sex differences (or lack of differences) were dependent on menstrual phase. Women were grouped by menstrual phase (EF, PO, or ML). EF and ML groups were combined (EF + ML) to compare menstrual phases characterized by low-moderate E2 (EF and ML) to a menstrual phase characterized by highest E2 (PO) levels. Performance was compared between menstrual phase groups and men using ANCOVA, including group as a between-subjects factor (three levels including men and two menstrual phase groups—EF + ML and PO), controlling for age. In case of a significant main effect of group (men, EF + ML, PO), multiple comparison corrected Tukey post hoc analyses were carried out to disentangle this effect. Effect size estimates (*η*^2^ or Cohen’s *d*) were calculated for all parametric analyses.

For comparisons of demographic variables, if model assumptions were violated, Mann–Whitney *U* tests or Kruskal–Wallis tests followed by post-hoc Dunn’s tests were conducted. For comparisons of behavioural variables, if model assumptions were violated, data was winsorized to the values at the 95th or 5th percentiles of the distribution. An alpha level of 0.05 was used for all statistical tests.

Data were analyzed to determine effects of sex and menstrual phase on navigation performance and self-reported strategy use. Dependent variables of interest were divided based on outcome domains of interest. For the posterior hippocampus-dependent cognitive map formation and topographical memory domain, dependent variables included fraction of correct recognition trials (accuracy) for the Four Mountains Task and number of trials needed to correctly remember the locations of all map landmarks for the Cognitive Map Task. For the anterior hippocampus- and entorhinal cortex-dependent face recognition domain, the dependent variables were overall accuracy and accuracy for each of three parts of the Cambridge Face Memory Test. For the entorhinal cortex-dependent path integration domain, dependent variables were absolute error (including combined angular and distance error from the target) for the Path and Location Integration Tasks. The dependent variable of interest for the domain of self-reported spatial navigation included scores on the Navigational Strategies Questionnaire, measuring propensity to report posterior hippocampus-dependent cognitive map-based navigation strategy use.

The original aim of this study was to address whether menstrual cycle would affect navigation; therefore, videogame experience was not included as a covariate for hypothesis-driven confirmatory analyses. Additional exploratory analyses were conducted including videogame experience (based on self-reported number of hours playing videogames per week) as a covariate in all models to explore whether menstrual phase effects were maintained above and beyond videogame experience. Results with the videogame experience covariate are reported in Supplementary Information (‘Results controlling for videogame experience’).

Once it was ascertained that face recognition and Location Integration Task performance fluctuated similarly across the menstrual cycle, an exploratory analysis correlating face recognition and location integration performance was conducted using Pearson correlation to investigate how these abilities related.

### Ethics approval

This study was performed in line with the principles of the Declaration of Helsinki. Approval was granted by the Ethics Committee of University of Toronto (Human Protocol Number 39691).

### Consent to participate

Informed consent was obtained from all individual participants included in the study.

### Supplementary Information

Supplementary Information.

## Data Availability

The data that support the findings of this study are available in the Open Science Framework, https://osf.io/nakd5.

## Code availability

The code used to analyze data for this study are available in the Open Science Framework, https://osf.io/nakd5.
