# Supplementary material for: Sex and menstrual cycle influence human spatial navigation strategies and performance
Source: Sci Rep. 2023 Sep 11;13:14953. doi: 10.1038/s41598-023-41153-x (PMC10495464; doi:10.1038/s41598-023-41153-x)
Supplement: Supplementary file 1 — Supplementary Information. [file 41598_2023_41153_MOESM1_ESM.docx]

**Supplementary Information**

**Sex and menstrual cycle influence human spatial navigation strategies and performance**

Alana Brown^1*^, Ford Burles^2^, Giuseppe Iaria^2^, Gillian Einstein^1,3,4#^, Morris Moscovitch^1,3#^

^*^Corresponding author

^#^Co-authors contributed equally to this work

^1^Psychology, University of Toronto, Toronto, Ontario, Canada M5S 3G3, ^2^Department of Psychology, Hotchkiss Brain Institute, and Alberta Children’s Hospital Research Institute, University of Calgary, Calgary, Alberta, Canada, T2N 1N4, ^3^Rotman Research Institute, Baycrest Health Sciences, Toronto, Ontario, Canada M6A 2E1, ^4^Linköping University, Linköping, Sweden 581 83

**Corresponding Author**

Alana Brown, **Email:**  [alana.brown@mail.utoronto.ca](mailto:alana.brown@mail.utoronto.ca), **Postal address:** 100 St. George Street, Toronto, ON M5S 3G3, Canada, **ORCID:** 0000-0002-1237-1314

**Supplementary Information**

**Supplementary Table S1**

*Summary: Excluded Participants*

| Exclusion Reason | Excluded *n* |
| --- | --- |
| Reproductive inclusion criteria not met (e.g., polycystic ovarian syndrome, endometriosis, fibroids, endometrial ablation, tubal ligation, autoimmune disorder affecting menstrual cycling, hysterectomy, and/or oophorectomy) | 23 |
| Pregnant currently or within past six months | 2 |
| Breastfeeding currently or within past six months | 15 |
| Taking birth control currently or within past six months | 50 |
| Spontaneously postmenopausal | 17 |
| Perimenopausal | 8 |
| Irregular menstrual cycling/short or long cycle length (outside range of 21-35 days) | 12 |
| Menstrual cycle/grouping could not be determined based on participant answers | 37 |
| Failed attention checks distributed throughout study (mathematical equations and text entry responses) | 5 |
| Technical issues without completion of any tasks | 2 |
| Neurological condition (e.g., electroconvulsive therapy resulting in seizures and migraines, stroke, schizophrenia, dementia) | 8 |
| Repeated study attempts | 9 |
| Above or below age 18-45 years | 16 |
| Below age 25 years | 7 |
| Further age matching by group: |  |
| Men: | 9 |
| Women EF: | 3 |
| Women PO: | 7 |
| Women ML: | 10 |
| Total excluded | 240 |

**Age-Related Participant Exclusion Details**

The current study planned to include participants aged 18-45 years. Sixteen participants were excluded for being over age 45 years. Men were significantly younger than all groups of women; therefore, the age cut-off was narrowed to age 25-45 years. Seven participants were excluded for being below age 25 years (all men). After these exclusions, men were still significantly younger than all groups of women. Thus, the youngest men and oldest women in the EF, PO, and ML groups were randomly excluded until the significant age differences between menstrual groups and men disappeared. This involved excluding nine men aged 25-27 years, three EF women aged 44-45 years, 10 ML women aged 44-45 years, and seven PO women aged 43-45 years.

**Menstrual Cycle Phase Categorization**

The following questions were asked online to estimate menstrual cycle phase:

1. When was the first day of your last menstrual period (approximate month/day/year)?
2. On average, how many days is your full menstrual cycle (e.g., how many days between the start of one period until the day before the next)? Note: the average cycle length is 28 days.
3. How many days do you typically menstruate (e.g., how many days of menstrual bleeding do you experience)?
4. Which day of your menstrual cycle are you currently on (assuming the first day of your period (menstrual bleeding) is Day 1)?
5. Have you gotten your period in the past 7 days?
6. When did your most recent period begin? Options: Between 1 and 2 days ago, between 3 and 5 days ago, between 6 and 7 days ago, more than 7 days ago
7. When do you expect your next period will begin? Options: Sometime in the next 6 days, between 7 and 10 days from now, not for 11 days or more, not sure
8. Has your cycle been regular (e.g., occurring consistently every 24-35 days) in the past 6 months?

For these questions, participants were asked to indicate the confidence they had in their estimations (options included: definitely accurate, probably accurate, might not be accurate, or definitely not accurate). Participants were excluded if they indicated any of their answers were “definitely not accurate”. We followed up with participants via email when it was not possible to calculate their current menstrual phase based on answers to menstrual cycle-related questions. If a participant did not reply to emails, they were excluded from analyses.

**Task-Related Data Quality Assessment**

Data quality control measures were used to exclude participants completing the Four Mountains Task and Cambridge Face Memory Test. A multivariate model was used to identify accuracy and performance outliers at the participant level, with the specific aim to identify participants responding without attempting to perform the task. A quality score was used to estimate how unlikely a participant’s accuracy and reaction time were compared to the whole cohort. This was used to identify participants with low accuracy and atypically fast reaction times, which was interpreted as indicating they were guessing without effort.

For quality control metrics, the following was generated for the Four Mountains Task and Cambridge Face Memory Test: z) a cumulative binomial distribution based on the inverse of the probability of receiving any given accuracy score based on guessing alone (i.e., the probability the participant was not guessing randomly). The following steps were then completed:

1. A variable representing the likelihood the accuracy score was 'bad' was produced by computing 1-(z^2^).
2. A weight for the participant’s datapoint was produced by squaring the 'probability the participant was not guessing randomly'.
3. The weighted percentile rank (c) was computed based on the participant's median reaction time (across the entire dataset, weights were based on accuracy)
4. The weighted percentile rank was rescaled to be between -1 and 1. This was done with the following equation: d=2c-1.
5. The positive tail of (d) was compressed by cubing it with the following equation: If(d>0), e = d^3^; else e=d. This was done because long reaction times are not as concerning as extremely short reaction times.
6. (e) was then squared.
7. (a) or (f) was selected, depending on which was smaller.
8. The final quality control score was produced with the following equation:1-g.

**Demographic Data**

**Demographic Data: Comparing Men and Women**

The Q-Q plots for men and women for number of years of education and anxiety severity (General Anxiety Disorder-7 Scale) were skewed; therefore, non-parametric Mann-Whitney U tests were used. Years of education (*U*=3691, *p*=0.60) and anxiety severity (*U*=3787, *p*=0.41) did not differ significantly between women and men. Normality assumptions were met for reported depressive symptoms (Center for Epidemiological Studies-Depression Scale); therefore, a parametric one-sample t-test was used to compare women and men. Reported depressive symptoms did not differ significantly between women and men (*t*(181)=1.18, *p*=0.24, *d*=0.19).

**Demographic Data: Comparing Men, EF+ML, and PO Women**

Q-Q plots for number of years of education were skewed; therefore, a non-parametric Kruskal-Wallis test was used to compare groups. Years of education did not differ significantly between men, EF+ML, and PO (*χ^2^*=2.07, *p*=0.36). ANOVA showed there was not a significant effect of group on anxiety severity (*F*(2,180)=0.49, *p*=0.61, *η*^2^=0.01) and depressive symptoms (*F*(2,180)=0.73, *p*=0.48, *η*^2^=0.01).

**Results Controlling for Videogame Experience**

Additional exploratory analyses controlling for videogame experience (self-reported number of hours playing videogames per week) were conducted to investigate whether sex- and menstrual cycle-dependent effects were maintained above and beyond variation in videogame experience.

**Four Mountains and Cognitive Map Tasks (Presumably Posterior Hippocampus-Dependent)**

***Sex Effects: Comparing Women and Men***

Analyses of covariance (ANCOVAs) controlling for age and videogame experience were used to compare women and men on Four Mountains Task accuracy and number of trials to reach criterion on the Cognitive Map Task. There was not a significant main effect of sex on Four Mountains Task accuracy (*F*(1,166)=0.11, *p*=0.74, partial *η*^2^=0.001) or on number of trials to reach criterion on the Cognitive Map Task (*F*(1,169)=0.003, *p*=0.96, partial *η*^2^=0.00002).

***Menstrual Phase Effects: Comparing Men, EF+ML, and PO Women***

ANCOVAs controlling for age and videogame experience revealed there was not a significant main effect of group on Four Mountains Task accuracy (*F*(2,165)=0.11, *p*=0.89, partial *η*^2^=0.001) or number of trials to reach criterion on the Cognitive Map Task *F*(2,168)=0.06, *p*=0.94, partial *η*^2^=0.001).

**Cambridge Face Memory Test (Presumably Anterior Hippocampus- and Entorhinal Cortex-Dependent)**

***Sex Effects: Comparing Women and Men***

ANCOVA controlling for age and videogame experience was used to compare women and men on overall accuracy for the Cambridge Face Memory Test. Women performed significantly better than men on overall accuracy (*F*(1,179)=4.20, *p*=0.042, partial *η*^2^=0.02).

Exploratory analyses were conducted to assess which task parts were driving the significant sex difference. ANCOVA controlling for age and videogame experience revealed there was not a significant main effect of sex on accuracy during Part 1 (*F*(1,179)=0.07, *p*=0.80, partial *η*^2^=0.0004). For the more challenging holistic processing-dependent component of the task (recognizing the studied face from different angles and/or lighting conditions; Part 2), ANCOVA controlling for age and videogame experience revealed women performed significantly better than men (*F*(1,179)=4.50, *p*=0.035, partial *η*^2^=0.02). During the most difficult part of the task, thought to be most dependent on holistic processing and the entorhinal cortex (recognizing the studied face from a different angle with overlaid visual noise; Part 3), ANCOVA controlling for age and videogame experience revealed there was not a significant main effect of sex on accuracy (*F*(1,179)=2.05, *p*=0.15, partial *η*^2^=0.01).

***Menstrual Phase Effects: Comparing Men, EF+ML, and PO Women***

ANCOVA controlling for age and videogame experience revealed a significant main effect of group on overall face recognition accuracy (*F*(2,178)=4.87, *p*=0.009, partial *η*^2^=0.05). *Post hoc* comparisons showed PO outperformed men (*t*(178)=-3.04, *p_Tukey_*=0.008, *d*=-0.63). There was a trend toward PO significantly outperforming EF+ML (*t*(178)=-2.33, *p_Tukey_*=0.054, *d*=-0.43). There were no significant overall accuracy differences between EF+ML and men (*t*(178)=-1.11, *p_Tukey_*=0.51, *d*=-0.20).

Exploratory analyses were conducted to assess which parts of the task were driving significant group differences. ANCOVA controlling for age and videogame experience showed there was not a significant main effect of group on Part 1 accuracy (*F*(2,178)=0.98, *p*=0.38, partial *η*^2^=0.01; Supplementary Fig. S1A). ANCOVA controlling for age and videogame experience revealed there was a significant main effect of group on Part 2 accuracy (*F*(2,178)=4.05, *p*=0.02, *η*^2^=0.04; Supplementary Fig. S1B). *Post hoc* comparisons showed PO outperformed men (*t*(178)=-2.83, *p_Tukey_*=0.01, *d*=-0.59). For Part 2 accuracy, there were no significant differences between EF+ML and PO (*t*(178)=-1.88, *p_Tukey_*=0.15, *d*=-0.35) and EF+ML and men *t*(178)=-1.33, *p_Tukey_*=0.38, *d*=-0.24). ANCOVA controlling for age also showed a significant main effect of group on Part 3 accuracy (*F*(2,178)=4.18, *p*=0.02, partial *η*^2^=0.04; Supplementary Fig. S1C). *Post hoc* comparisons showed PO significantly outperformed EF+ML (*t*(178)=-2.50, *p_Tukey_*=0.036, *d*=-0.46) and men (*t*(178)=-2.63, *p_Tukey_*=0.03, *d*=-0.55). For Part 3 accuracy, there were no significant differences between EF+ML and men (*t*(178)=-0.47, *p_Tukey_*=0.89, *d*=-0.09).

**Location and Path Integration Tasks (Presumably Entorhinal Cortex-Dependent)**

***Sex Effects: Comparing Women and Men***

For the Location Integration Task, ANCOVA controlling for age and videogame experience showed there was not a significant main effect of sex on absolute error (*F*(1,177)=3.46, *p*=0.064, partial *η*^2^=0.02).

For the Path Integration Task, ANCOVA controlling for age and videogame experience showed there was not a significant main effect of sex on absolute error (*F*(1,177)=3.30, *p*=0.07, partial *η*^2^=0.02).

***Menstrual Phase Effects: Comparing Men, EF+ML, and PO Women***

For the Location Integration Task, ANCOVA controlling for age and videogame experience showed there was a significant main effect of group on absolute error (*F*(2,176)=4.17, *p*=0.017, *η*^2^=0.05); *post hoc* comparisons showed PO made smaller errors than men (*t*(176)=2.80, *p_Tukey_*=0.015, *d*=0.59) and trended toward making smaller errors than EF+ML (*t*(176)=2.19, *p_Tukey_*=0.075, *d*=0.41). There were no significant error differences between EF+ML and men (*t*(176)=0.98, *p_Tukey_*=0.59, *d*=0.18).

For the Path Integration Task, ANCOVA controlling for age and videogame experience showed there was not a significant main effect of group on absolute error (*F*(2,176)=2.31, *p*=0.10, *η*^2^=0.03).

**Navigational Strategies Questionnaire**

***Sex Effects: Comparing Women and Men***

ANCOVA controlling for age and videogame experience showed there was a significant main effect of sex on scores for the Navigational Strategies Questionnaire (*F*(1,179)=9.38, *p*=0.003, partial *η*^2^=0.05); compared to women, men demonstrated increased tendency to report use of posterior hippocampus-dependent map-based navigation strategies. Compared to men, women demonstrated increased tendency to use scene-based strategies.

***Menstrual Phase Effects: Comparing Men, EF+ML, and PO Women***

ANCOVA controlling for age and videogame experience showed there was a significant main effect of group on scores for the Navigational Strategies Questionnaire (*F*(2,178)=4.73, *p*=0.01, partial *η*^2^=0.05); *post hoc* comparisons showed men reported significantly increased use of map-based strategies compared to PO (*t*(178)=2.70, *p_Tukey_*=0.02, *d*=0.56) and EF+ML (*t*(178)=2.73, *p_Tukey_*=0.02, *d*=0.50). For Navigational Strategies Questionnaire scores, there was not a significant difference between EF+ML and PO (*t*(178)=0.35, *p_Tukey_*=0.93, *d*=0.06).

**Supplementary Fig. S1** Barplots depicting sex and menstrual phase means and comparisons for Cambridge Face Memory Task Performance for Parts 1-3

*
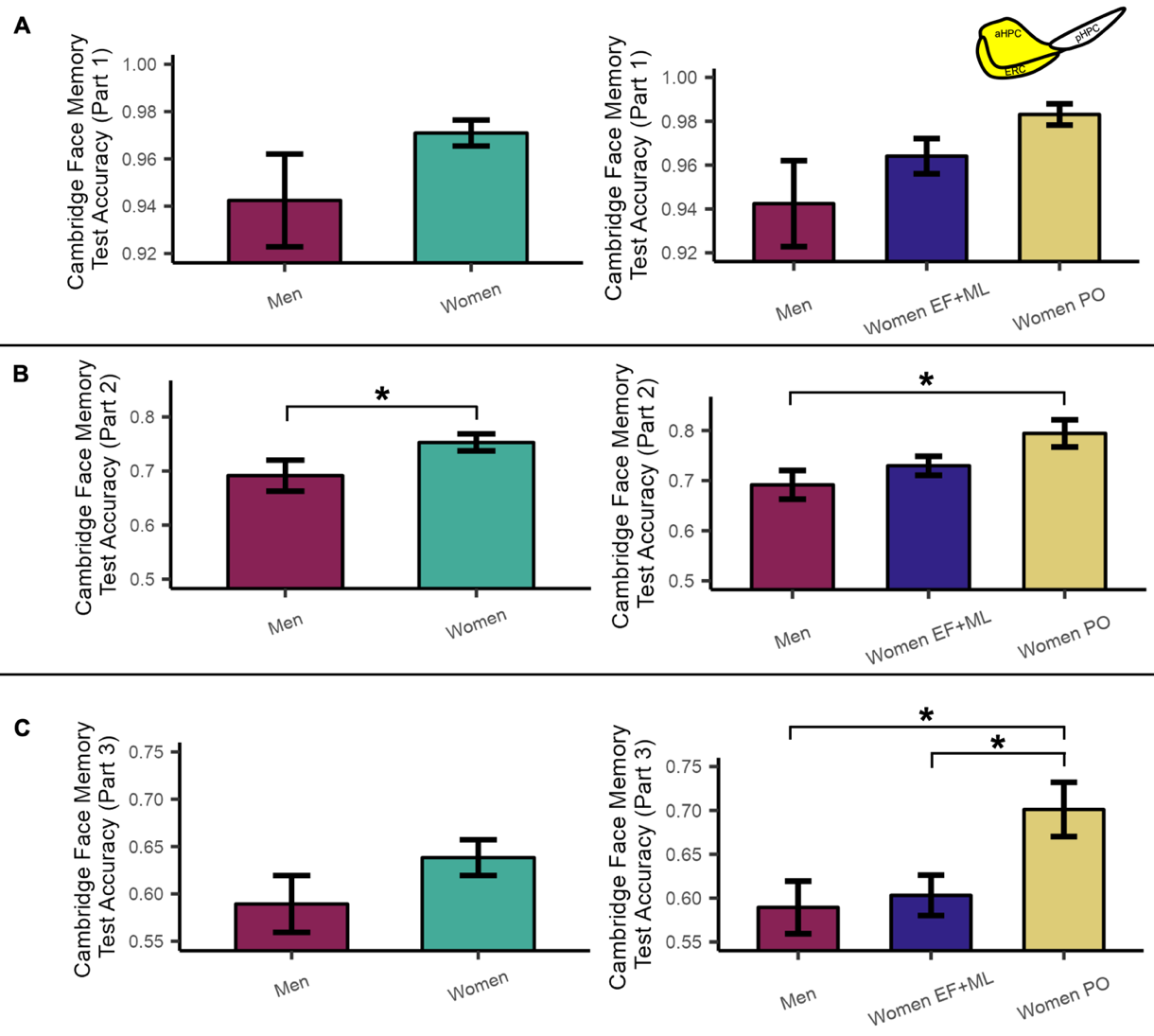
*

**Note:** A) Cambridge Face Memory Test Part 1 accuracy; B) Cambridge Face Memory Test Part 2 accuracy; C) Cambridge Face Memory Test Part 3 accuracy. Yellow highlight=medial temporal lobe region that may be particularly relevant for the measure. **Abbreviations:** PO=late follicular/periovulatory menstrual phase; ML=mid/late luteal menstrual phase; EF=early follicular menstrual phase. Error bars represent standard error of the mean. * = *p*<0.05.

**Supplementary Fig. S2** *Barplots depicting sex and menstrual phase means and comparisons when controlling for videogame experience for task and questionnaire measures*


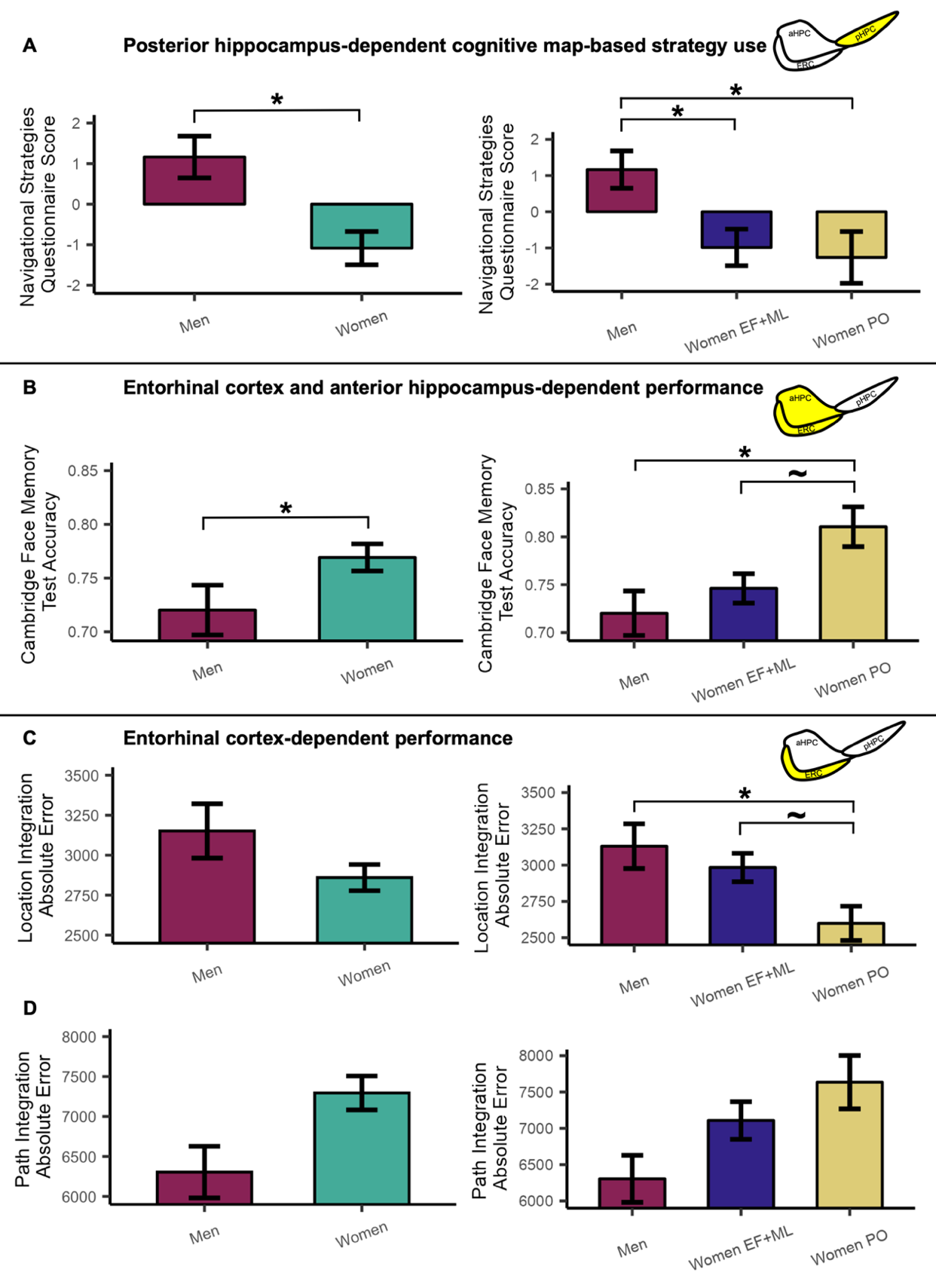


**Note:** A) Navigation Strategies Questionnaire score; B) Cambridge Face Memory Test accuracy; C) Location Integration Task absolute error; D) Path Integration Task absolute error. Yellow highlight=medial temporal lobe region that may be particularly relevant for the measure. **Abbreviations:** PO=late follicular/periovulatory menstrual phase; ML=mid/late luteal menstrual phase; EF=early follicular menstrual phase; aHPC=anterior hippocampus; pHPC=posterior hippocampus; ERC=entorhinal cortex. *=*p*<0.05; ~=*p*<0.08.

**All Menstrual Phase Effects: Results Comparing Men, EF, PO, and ML Women**

**Four Mountains and Cognitive Map Tasks (Presumably Posterior Hippocampus-Dependent)**

For comparisons of EF, PO, ML, and men, Levene’s Test showed the assumption of homogeneity of variance was not met; therefore, Four Mountains Task accuracy data was winsorized to the values at the 95^th^ or 5^th^ percentiles of the distribution.

ANCOVAs controlling for age revealed there was not a significant main effect of group on Four Mountains Task accuracy (*F*(3,165)=0.15, *p*=0.93, partial *η*^2^=0.003) or number of trials to reach criterion on the Cognitive Map Task *F*(3,168)=0.18, *p*=0.91, partial *η*^2^=0.003).

**Cambridge Face Memory Test (Presumably Anterior Hippocampus- and Entorhinal Cortex-Dependent)**

ANCOVA controlling for age revealed a significant main effect of group on overall face recognition accuracy (*F*(3,178)=3.20, *p*=0.025, partial *η*^2^=0.05). *Post hoc* comparisons showed PO outperformed men (*t*(178)=-3.00, *p_Tukey_*=0.016, *d*=-0.61). There were no other significant group differences on overall face recognition accuracy.

**Location and Path Integration Tasks (Presumably Entorhinal Cortex-Dependent)**

For comparisons of EF, PO, ML, and men, Levene’s Test showed the assumption of homogeneity of variance was not met; therefore, Location Integration Task absolute error data was winsorized to the values at the 95^th^ or 5^th^ percentiles of the distribution.

For the Location Integration Task, ANCOVA controlling for age showed there was a significant main effect of group on absolute error (*F*(3,176)=3.02, *p*=0.03, *η*^2^=0.05); *post hoc* comparisons showed PO made smaller errors than men (*t*(176)=2.79, *p_Tukey_*=0.03, *d*=0.57) and trended toward making significantly smaller errors than EF (*t*(176)=2.34, *p_Tukey_*=0.092, *d*=0.50). There were no other significant group differences.

For the Path Integration Task, ANCOVA controlling for age showed there was not a significant main effect of group on absolute error (*F*(3,176)=2.28, *p*=0.08, *η*^2^=0.04).

**Navigational Strategies Questionnaire**

ANCOVA controlling for age showed there was a significant main effect of group on scores for the Navigational Strategies Questionnaire (*F*(3,178)=3.62, *p*=0.01, partial *η*^2^=0.06); *post hoc* comparisons showed men reported significantly increased use of map-based strategies compared to PO (*t*(178)=2.86, *p_Tukey_*=0.02, *d*=0.58) and trended toward reporting significantly increased use of map-based strategies compared to EF (*t*(178)=2.33, *p_Tukey_*=0.09, *d*=0.48) and ML (*t*(178)=2.59, *p_Tukey_*=0.051, *d*=0.54). For Navigational Strategies Questionnaire scores, there were no significant differences between EF, ML, and PO.
